# Patient experiences with depression care in general practice: a qualitative questionnaire study

**DOI:** 10.1080/02813432.2022.2074069

**Published:** 2022-05-23

**Authors:** Ina Grung, Norman Anderssen, Inger Haukenes, Sabine Ruths, Tone Smith-Sivertsen, Øystein Hetlevik, Stefan Hjørleifsson

**Affiliations:** a Research Unit for General Practice, NORCE Norwegian Research Centre, Bergen, Norway; b Department of Global Public Health and Primary Care, University of Bergen, Bergen, Norway; c Department of Psychosocial Science, University of Bergen, Bergen, Norway; d Division of Psychiatry, Haukeland University Hospital, Bergen, Norway

**Keywords:** Depression, general practice, patient perspective, primary health care, Norway

## Abstract

**Objective:**

To investigate patient experiences and preferences regarding depression care in general practice.

**Design and setting:**

A qualitative study based on free-text responses in a web-based survey in 2017. Participants were recruited by open invitation on the web page of a Norwegian patient organization for mental health. The survey consisted of four open-ended questions concerning depression care provided by general practitioners (GPs), including positive and negative experiences, and suggestions for improvement. The responses were analysed by Template Analysis.

**Subjects:**

250 persons completed the web-based survey, 86% were women.

**Results:**

The analysis revealed five themes: The informants appreciated help from their GP; they wanted to be met by the GP with a listening, accepting, understanding and respectful attitude; they wanted to be involved in decisions regarding their treatment, including antidepressants which they thought should not be prescribed without follow-up; when referred to secondary mental care they found it wrong to have to find and contact a caregiver themselves; and they thought sickness certification should be individualised to be helpful.

**Conclusions:**

Patients in Norway appreciate the depression care they receive from their GP. It is important for patients to be involved in decision-making regarding their treatment.

**KEY POINTS**
Depression is common, and GPs are often patients’ first point of contact when they seek help.  • Patients who feel depressed appreciate help from their GP.  • Patients prefer an empathetic GP who listens attentively and acknowledges their problems.  • Individualised follow-up is essential when prescribing antidepressants, making a referral, or issuing a sickness absence certificate.

## Introduction

Depression is among the three leading causes of years lived with disability, with huge societal and personal consequences across gender and age groups [1]. In Norway, an estimate suggests that every 5th person will suffer from depression at some point in life [2], and depression is the second most important contributor to health loss in the population.

When people need help for symptoms of depression, the GP is often the first professional they seek, and the management of patients with such problems represents an important task for GPs. In Norway, each year 3–4% of the population have one or more consultations with a GP where depression or depressive symptoms are reported by the GP to be the main reason for the contact, and depression is the sixth most frequent diagnosis in general practice consultations [3].

A review of European community studies estimated that only 26% of people with mental disorders seek health care [4], and those who seek help are not always those with the most severe symptoms [5,6]. However, the total prevalence of depression is so high that those who seek help from their GP are still many, and the severity of their symptoms ranges from sub-threshold, mild, moderate to severe depression. In this article we will refer to all of the above as “depressed patients”.

According to earlier international studies more than 90% of patients consulting for depression were treated by GPs alone [7]. Today, 20% of patients with a new diagnosis of depression in Norway are referred to specialised care [8]. In addition, an increasing proportion of patients probably seeks help from private therapists or from community-based low-threshold services, although there is no official statistics on the extent to which these services are being used. Nevertheless, it is reasonable to assume that a large majority of patients who seek help for depression are still being treated by GPs [4,9]. This is in line with Norwegian guidelines promoting a stepped-care approach, i.e. treating the patient at the lowest effective level possible, and referring to secondary care only when treatment in primary care is not sufficient [2].

GP depression care can consist of different measures including talking therapy, help with problem-solving, medication, sick listing and referral to secondary care depending on the severity and patient preferences [10,11]. In a Danish study [12], features of general practice such as open receptiveness, long-term doctor-patient relationships and prior knowledge of the patient’s history were essential in GPs’ depression care. Regarding talking therapy, GPs can combine components from different psychological methods [12]. According to a recent Norwegian study, GPs prescribed an antidepressant medication to 31% of patients during the first 12 months of a new episode of depression [13], and another Norwegian study found that 44% of patients with depression received a sickness certificate [14].

In Norway, GPs act as gatekeepers for secondary care. A referral from a GP is thus required for patients to receive refundable psychotherapy or psychiatric treatment [15]. It is crucial to have knowledge about patient experiences and preferences when evaluating care and identifying areas in need of improvement [16]. Positive patient experiences are associated with improved safety, adherence to treatment, clinical effectiveness, and health outcomes [17]. According to a Norwegian study among patients in GP waiting rooms on the preferences of patients who had suffered from depression, 61% would prefer talking therapy with their GP in the case of future depression, 53% referred to a psychologist or psychiatrist, and 23% medication [18]. This is in line with a meta-analysis of studies that showed a 70% greater patient preference for psychological treatment than for pharmaceutical treatment for depression [19]. Another Norwegian study has shown that depressed patients experience fragmented care and a lack of user involvement in important decisions [20]. Apart from this, there is limited knowledge about how patients experience depression care in Norwegian general practice.

In sum, while GPs in Norway perform and are expected to perform a central role in depression care, there is limited knowledge about how patients experience the help they receive from their GPs and their preferences. The aim of the current study was therefore to investigate the experiences and preferences of patients regarding depression care in Norwegian general practice.

## Material and methods

We conducted a qualitative survey using an anonymous web-based questionnaire in a nation-wide convenience sample. Participants were recruited through the web page of «Mental Health», (https://mentalhelse.no), a Norwegian organization promoting mental health, the members being persons with mental illness, next-of-kin and other interested persons. The invitation read: “Management of depressive symptoms in primary care: Please participate in a brief survey from Uni Research Health and the University of Bergen” (see appendix 1). Those who followed the initial link were guided to the more extensive information about the study (see appendix 1), and if they provided an affirmative response to the question “Have you ever told your general practitioner that you felt depressed?” they were invited to respond to four open-ended questions:

1. What did your GP do that helped you?
2. What did the GP do that was not of much help?
3. What do you think can be done so that patients with depression will get more help from their GP?
4. What worked and did not work in the GPs cooperation with others (i.e. your psychologist, psychiatrist, physiotherapist, social services, or employer)?

The participants were asked about their gender (male, female), age (five categories from 12 years of age) and educational level (three categories). Data were collected during an eight-day period in August 2017, with the largest number of responses on day one, before the number of responses gradually decreased day by day. After the first days of recruitment, we adjusted the invitation text to encourage more men to participate (see Appendix 1), as most participants so far were women. After eight days we considered the number of responses to be sufficient. The research team included four GPs, a social psychologist, a physiotherapist and philosopher, and a psychiatrist.

### Analysis

The written responses to the open-ended questions were subjected to Template Analysis [21], a qualitative method that makes use of hierarchical coding themes and subthemes. Template Analysis has been developed to explore text materials such as interview transcripts. It resembles other text condensation procedures [22,23] but with a higher degree of tentative predefined content structure, while also having the flexibility to adapt to the needs of the study. Template Analysis is well suited for studies with many responses.

The analysis started with authors IG, SH and NA reading through all the responses to get an overall impression. Second, IG in collaboration with SH and NA identified units of meaning, and these were given tentative labels. IG then grouped all units of meaning into tentative main categories and sub-categories. These were repeatedly discussed in detail with the co-authors before a final coding structure was decided upon. A draft was made by the first author and shared with the rest of the authors for further discussion and feedback, and then the article was re-evaluated again before it came to its final form.

### Study sample

Altogether 250 participants completed the survey, 86% were women. We have no information on those who did not complete the survey. Most participants were aged 35–50 years (44%) or 19–34 years (35%), while 16% were 51–66 years. Only 3% of the participants were in the age group 12–18 and 2% 67–74 years. 7% of participants had completed primary and lower secondary school, 39% senior high school (typically 16–19 years of age), and 54% university or university college.

## Results

250 responses were generated, describing first-hand experiences and preferences regarding GP depression care. The numbers of responses to the four open-ended questions were 248, 235, 246 and 228, respectively. The shortest response was one word, the longest 153 words, with most responses in the range of 4–30 words. Some of the responses indicated a mismatch between the help the informants had experienced and their preferences, and it was clear that many of the informants would prefer to be more involved in decisions regarding their treatment. The responses included descriptions of GPs having helped the informants through a broad range of interventions, from advice about physical activity and close follow-up in consultations to sickness certification and referral to specialist care, even including admittance to a psychiatric ward. Through the coding, sorting, and synthesizing process across all responses and across the four questions, we identified five main themes.

### Theme 1: GP help and follow-up are valued

The main theme was an appreciation of receiving help from a GP, sometimes accompanied by a wish for more contact and help. Except for 14 informants who answered “nothing” or left the question blank, all reported that their GP had helped them.

I was lucky to have a helping GP (female, 35–50 years old, university or university college).

My GP is fantastic (female, 51–66 years old, senior high school).

Many wanted their GP to be more involved, even when they were seeing a therapist in secondary care.

Follow-up is extremely important (female, 35–50 years old, senior high school).

Offered me a double session after only a few days (female, 19–34 years old, senior high school).

The type of help received, and the informants’ judgements about what had been most helpful and what help they would like to receive in the future varied. This will be elaborated on in the following themes.

### Theme 2: Attentive listening and acknowledgement is essential

The informants stressed that the GP should listen carefully and acknowledge depression as an illness worthy of medical attention. Many different phrases related to attentive listening and acceptance were used, indicating that the informants found it important for the GP to acknowledge the struggle of their patients and their difficult situations. The informants typically wanted a caring, supporting, respectful and empathic doctor, a doctor who would let them talk about their problems, and a doctor who would take the time to listen with acceptance. Having a GP listen carefully to your concerns, was the fundamental experience conveyed by many informants.

He took the time to talk to me and didn’t care if there were many people in the waiting room (female, 51–66 years old, university or university college).

He took the time to listen to what I had to say (female, 35–50 years old, university or university college).

The informants wished for their GP to show interest in what was causing their ailments because they thought this was necessary for the GP to help properly. Many wanted the doctor to show interest in their life and their problems, as stated below.

We need to talk about the reasons why, and the triggering factors of the depression (female, 35–50 years old, university or university college).

I felt that the GP was interested in my life (female, 35–50 years old, university or university college).

Also, to feel acknowledged by their GP when presenting with depression was emphasized.

GPs must acknowledge psychological illness (female, 19–34 years old, senior high school).

Take the patient seriously from day one. It takes a lot to take the step and actually admits that you are struggling, and may have struggled for years (male, 19–34 years old, senior high school).

Understanding and knowledge about what it is like to be depressed. Professional care and empathy (female, 35–50 years old, university or university college).

### Theme 3: Antidepressants should not be the only treatment

Circumstances regarding drug prescription emerged as crucial and the importance of following up with the patient if antidepressants were prescribed, was emphasised. Although many informants had experienced the prescription of antidepressants as help from their GP, when describing their preferences for future treatment most informants only mentioned antidepressants in combination with other initiatives. Several informants conveyed that it was hard to trust the doctor if he or she prescribed medications without making him-herself thoroughly acquainted with the patient’s situation. Antidepressants should never be prescribed without simultaneous talking therapy, was stated.

Many doctors prescribe antidepressants and think that everything will be alright. The truth is that antidepressants don’t solve it all (female, 19–34 years old, primary school).

Many informants stressed that they wanted to participate in the decision about whether antidepressants should be part of their treatment. Some stated that they did not like to take antidepressants at all, and some had felt that their GP had pressed them to take such medications or held it against them if they refused:

Also, I have met the prejudice that I don’t want to be helped when I don’t want to take strong medications that require a doctor’s prescription and that is vaguely documented (female, 19–34 years old, senior high school).

### Theme 4: Handling own referral is too much

How referrals were handled emerged as a main concern to the informants. Most informants who had asked for a referral received one, but the process around this was problematized, especially concerning follow-up by their GP. Some informants wrote that although the GP had provided the referral, they themselves had to find a psychotherapist. The GP just handed them a list of psychotherapists and asked them to choose one and try to get an appointment. The informants were not satisfied with this approach, as it was difficult to find the courage and energy to do this while struggling with depression. Also, the informants called for more help from the doctor in finding the right therapist. An informant explains the procedure:

Got a list with names of psychologists in my hand and had to fix the rest myself (female, 19–34 years old, university or university college).

Another aspect of the referral process was waiting time for access to the specialist. Many informants reported that receiving help took too long, and some thought the doctor should have suggested referral to a specialist at an earlier stage.

Even if the doctor does a good job and refers you, in most instances, you have to wait very long before you get help (female, 51–66 years old, university or university college).

She might have referred me to a psychologist earlier without me having to ask for it after such a long time (female, 19–34 years old, university or university college).

Some informants had experienced that the referral made by their GP was rejected by the secondary health care and that this added a feeling of hopelessness to their situation.

### Theme 5: Sickness certificates must be customized

Many informants stated that the handling of their sickness certificate was important to them, especially the need to individualise and tailor the use of sickness absence certificates. It was not uncommon for informants to mention sickness certification in response to the question about what their GP did that was of help, either as the main intervention or in combination with other measures:

Got a sick leave from my job which I couldn’t handle at that point (female, 19–34 years old, university or university college).

A pivotal aspect included the duration of the sickness absence. The informants’ preferences and expectations about the duration of sick leave varied considerably. Some experienced that it was stressful if the GP only gave them a sickness certificate for a short period, such as two weeks at a time. Others had experienced that long periods of sick leave could lead to less follow-up.

Asked me how much of a sickness certification I wanted and for how long. When I was depressed and already had a bad conscience for not being able to work, it didn’t help much having to make that decision myself (male, 19–34 years old, senior high school).

As evident from the statement above, asking patients what they wanted was not always perceived as helpful. On the other hand, some informants reported that they had been denied a sickness certification when they consulted their doctor with depression.

Was told depressed people couldn’t get sick leave. Were better off staying at work. Not wise when you work with children (female, 51–66 years old, university or university college).

## Discussion

### Principal findings

In this qualitative study among 250 persons in Norway who had consulted their GP due to depressive symptoms, almost all reported having received valuable help and follow-up from their GP. However, there was sometimes a mismatch between the experiences of the informants and their preferences. The informants in particular valued attentive listening and acknowledgement of their suffering, as well as being involved in decisions about their own treatment. Those who were positive for antidepressants highlighted that such treatment should always be accompanied by follow-up. Finally, the informants said that when referral to a psychotherapist was required, patients should not have to handle this referral themselves and that the use of sickness certificates should be tailored to each patient’s situation.

### Strengths and limitations

A strength of this study is that 250 individuals with first-hand knowledge of seeking and receiving care from their GP for self-reported depression conveyed their perspectives anonymously, and in their own words. The open-ended survey questions elicited positive as well as negative responses, demonstrating a substantial variety in patients’ experiences and preferences.

The material was large, and thus we had to choose what parts to focus on. We do not know whether the informants fulfilled the criteria for a depression diagnosis, and we did not collect information about the severity of the participants’ depression or other relevant aspects of their illness. We are thus unable to relate the informants’ responses to depression severity or other features of their illness or their situation. However, the wide range of responses about what the informants’ GP had done to help them indicate that the informants had suffered from anything between severe to mild and probably also sub-threshold depression. This is in accordance with the fact that GPs provide help to patients according to their complaints, and diagnostic thresholds or severity are not always of high relevance to person-centred management in primary care. The informants were self-selected, and most of them were highly educated women, and this should be taken into account when considering the meaning and relevance of the findings [24]. The fact that some of the authors are GPs may have influenced the analysis towards a more positive view of GP depression care.

### Findings in relation to other studies

The current study confirms that depressed patients emphasize the relationship between the GP and the patient. In the responses, there was a strong desire to be accepted, understood and respected; this relates to the need for tailored care and follow-up, and it adds to previous research findings that good listening and follow-up are fundamental for depression care [25,26]. The beneficial effects of practitioner empathy are well known in consultations with patients with mental health problems [27,28]. A listening doctor is essential, not only for building the GP-patient relationship but also for diagnosing and monitoring disease [29].

Our findings resonate well with the fundamentals of the patient-centred clinical method, an integrated clinical method that combines two fundamental tasks for clinicians; to understand the patient and to understand the disease [30]. This is consistent with the findings of a study from Scotland where it was shown that the patient-centred method is helpful and appreciated by patients suffering from depression [31].

The first theme in the responses was that GP help was much appreciated. Recent political and professional debates on the limited availability of psychotherapy in Norway have mostly ignored treatment by GPs. GP depression care is low-threshold professional help that is easily accessible to the patients and economically favourable to society, thanks to the publicly funded health care system in Norway [15]. The more GPs have the capacity and skills to provide help to depressed patients, the less pressure will there be for treatment in secondary mental healthcare [32]. Thus, GPs’ contribution to depression care might be better acknowledged by policymakers and possibly among GPs themselves.

Although almost all informants reported having been helped by their GP, what kind of help they described varied considerably, and there was sometimes a mismatch between their experiences and their preferences. Some informants clearly expressed that they had not been given sufficient opportunity to participate in decisions regarding their care. There is growing recognition of the value of patients’ perspectives, especially when it comes to mental health care [33]. Patients were previously regarded as passive recipients of health services. This, however, has changed dramatically over the past decades - from compliance (the patient does what the doctor decides), through adherence (the patient is an active partner agreeing on the recommendations given by health workers) to concordance (the patient is involved in decisions regarding their own health). The perspectives and resources of the patient herself are now expected to be part of both clinical healthcare and -research [34]. This is also strongly emphasized in the national treatment guideline for depression in Norway, which states that adequate treatment can only be given once the GP has familiarized herself with the particulars of the patient’s situation. The guideline emphasizes that depression affects all aspects of being human, not only biological and psychological measures, but also existential, social, societal, and cultural relations. Further, the guideline states that the caregivers must make patients feel respected and cared for, including active participation in their own treatment [2]. Similarly, the Norwegian Patient Rights Act states that the patient has the right to participate in decisions on how their health care is carried out, such as choosing between available treatment methods. However, our findings indicate that patients still may feel insufficiently involved in treatment planning and that their preferences have not been asked for or taken into account. Thus, there seems to be room for improvement in the way GPs meet their depressed patients.

According to a Norwegian study from 2019 and an Australian study from 2017 [25,35] on patient-reported depression treatment in general practice, many depressed patients prefer other forms of treatment than antidepressants. Our study supports these findings, and another Norwegian study found that the proportion of patients receiving antidepressant treatment decreased in the period 2009–2015 [8]. However, our study also suggests that one of the reasons patients do not want antidepressants is that they do not want such treatment in the absence of follow-up.

The capacity problems of secondary mental health care in Norway are well known. 30% of referrals to psychiatric centres are rejected, and the waiting time to see a psychologist or psychiatrist with a reimbursement contract is often unreasonably long [36–39]. However, the findings of the current study tell us that it is not only the long waiting time in itself that is experienced as difficult. Many informants complained about the GP handing them a referral and expecting them to find a therapist themselves, and conversely, they appreciated it when the GP referred them directly to a psychotherapist whom the GP knew and could recommend.

## Conclusion

The current study is based on first-hand experiences from 250 self-recruited individuals who had received help from their GP for depression and reveals important information on how patients experience depression care in general practice. Based on the findings we suggest the following advice for GPs caring for patients with depression, in line with the Norwegian depression guideline and principles of patient-centered clinical care:

- Know that patients appreciate help from their GP and want to be involved in decisions about their treatment.
- Give priority and time to empathetic listening.
- Individualize follow-up and explore the patient’s views, including when considering antidepressants.
- When a referral is needed, provide help until the patient has an appointment with a therapist.
- Customise sickness certification to the patient’s needs.

## Data Availability

The dataset generated and analysed during the current study is not publicly available due to confidentiality.

## Appendix 1

### Questionnaire on depression in general practice

Depression is a common problem. Many people contact their general practitioner to get help, but we do not know much about what help they receive, how the GP cooperates with other professionals, and what needs improvement. Therefore we wish to hear from you who have sought help at your GP for depression!

The survey is part of a research project on what characterises good help for patients with depressive symptoms.

We hope that you who have sought help from your GP for depression are willing to answer eight questions from us. We do not ask about your name or other personal information, except for your age, sex and educational level. We do not want information that could be used to identify you or other persons. We will not at any time have access to your IP- address (the unique address of your PC).

The research project is performed at Uni Research Health and The University of Bergen. It has been approved by the Regional Committee for Medical and Health Research Ethics. Stefan Hjørleifsson is responsible for the project, and questions can be directed to stefan.hjorleifsson@uib.no or telephone number 55586090. If you would like to join the survey, press START below.

- While answering, you can withdraw from the survey at any time.
- When you have given your answers and pressed FINISH, you can no longer go back.
- By pressing FINISH you give your approval to the use of your answers in the research project.

We thank you in advance for your help.

#### Adjustment

During the first 24 h after the survey was published on the web pages of Mental Helse (Mental Health) on the 15th of August 2017, we have received an overwhelming amount of responses. We thank all of you who have answered and thus helped us with the research!

Most of the respondents so far are women.

We hope that also you who are male and have sought help from your GP for depression would like to answer the eight questions in the survey. We do not ask about your name or other personal information, only your age, sex and educational level. We do not want information that could be used to identify you or other persons. We will not at any time have access to your IP- address (the unique address of your PC). Press here if you would like to participate in this survey or would like more information – either you are a man or a woman.
